# Recent Advances in Chemically-Modified and Hybrid Carrageenan-Based Platforms for Drug Delivery, Wound Healing, and Tissue Engineering

**DOI:** 10.3390/polym13111744

**Published:** 2021-05-26

**Authors:** Hamidreza Mokhtari, Shima Tavakoli, Fereshteh Safarpour, Mahshid Kharaziha, Hamid Reza Bakhsheshi-Rad, Seeram Ramakrishna, Filippo Berto

**Affiliations:** 1 Department of Materials Engineering, Isfahan University of Technology, Isfahan 84156-83111, Iran; mokhtarirezahamid@gmail.com (H.M.); fereshtehsafarpour@yahoo.com (F.S.); 2 Division of Polymer Chemistry, Department of Chemistry-Ångstrom Laboratory, Uppsala University, SE75121 Uppsala, Sweden; shima.tavakoli@kemi.uu.se; 3 Advanced Materials Research Center, Department of Materials Engineering, Najafabad Branch, Islamic Azad University, Najafabad, Iran; 4 Department of Mechanical Engineering, National University of Singapore, 9 Engineering Drive 1, Singapore 117576, Singapore; seeram@nus.edu.sg; 5 Department of Mechanical and Industrial Engineering, Norwegian University of Science and Technology, 7491 Trondheim, Norway

**Keywords:** carrageenan, chemical modification, tissue engineering, wound healing, drug delivery

## Abstract

Recently, many studies have focused on carrageenan-based hydrogels for biomedical applications thanks to their intrinsic properties, including biodegradability, biocompatibility, resembling native glycosaminoglycans, antioxidants, antitumor, immunomodulatory, and anticoagulant properties. They can easily change to three-dimensional hydrogels using a simple ionic crosslinking process. However, there are some limitations, including the uncontrollable exchange of ions and the formation of a brittle hydrogel, which can be overcome via simple chemical modifications of polymer networks to form chemically crosslinked hydrogels with significant mechanical properties and a controlled degradation rate. Additionally, the incorporation of various types of nanoparticles and polymer networks into carrageenan hydrogels has resulted in the formation of hybrid platforms with significant mechanical, chemical and biological properties, making them suitable biomaterials for drug delivery (DD), tissue engineering (TE), and wound healing applications. Herein, we aim to overview the recent advances in various chemical modification approaches and hybrid carrageenan-based platforms for tissue engineering and drug delivery applications.

## 1. Introduction

Carrageenans are natural linear polysaccharides (carbohydrates) that are extracted from certain species of the class Rhodophyceae (red seaweeds). The most well-known and still most important red seaweed used for manufacturing the hydrophilic colloids to produce carrageenan is Chondrus crispus, which is a dark-red parsley-like plant that grows attached to the rocks [1]. Carrageenans primarily include changing 3-linked b-d-galac- topyranose (G-units), 4-linked a-d-galactopyranose (D-units), or 4-linked 3,6-anhydro-a-d-galactopyranose (DA-units), creating the disaccharide continuing unit of carrageenans [2]. In general, carrageenan is a sulfated polygalactan with 15–40% ester sulfate content, which makes it an anionic polysaccharide, and it can be mainly categorized into the following three different classes based on their sulfate contents: iota (ι)-, kappa (κ)-, and lambda (λ)-carrageenan [3]. The κ-, ι- and λ-carrageenans have one, two and three sulfate ester groups, in order, resulting in approximate correspondent calculated sulfate contents of 20%, 33% and 41% (*w*/*w*), respectively, although considerable variations can occur owing to the differences between the seaweed species or batches [4]. Higher levels of ester sulfate groups eventuate in lower gel strength and lower solubility temperature [5]. In this regard, κ- and ι- carrageenan have demonstrated similar properties as they can undergo a thermos-reversible conformational arrangement at higher temperatures, and at lower temperatures they can undergo network formation and combination through sulfate groups and the 3,6-anhydro-d-galactopyransyl ring. Additionally, they have shown gelling propertiesy in the presence of cations (i.e., K^+^ and Ca^2^) and their gel–sol transition temperatures heavily depend on the ion contents [6]. On the other hand, λ-carrageenan does not display any kind of 3, 6-anhydride bridge, opposite to κ and ι- carrageenan. Furthermore, it is incapable of generatingea gel, and possesses an arbitrary coil conformation at entire temperatures [3,5,7].

Carrageenans have been vigorously used in the food industry due to their distinguished physical and functional properties, such as stabilizing, thickening, and gelling abilities [2]. Besides, carrageenans are utilized in numerous non-food items and applications, including pharmaceutical, cosmetics, and printing. They have also shown several potential pharmaceutical activities such as antioxidant and antitumor, immunomodulatory, anti-hyperlipidemic, and anticoagulant properties [8]. Additionally, carrageenans were employed to synthesize carrageenan-based hydrogels for various bio-applications including tissue engineering (TE) and drug delivery (DD) systems and regenerative medicine [9]. However, these applications have been limited due to the difficulty of controlling gelation properties, stability, and degradation rate in the physiological environment [10]. Various physical or chemical crosslinking methods allow the formation of networks with better properties to overcome these challenges. In physical crosslinking, the network is fabricated through the formation of physical bonds between the different polymer chains [11]. Studies have shown that the presence of KCl during the ionic-crosslinking process leads to the formation of hydrogels with strong interplays between the ion and sulfate groups. The ionic crosslinking process promotes the mechanical strength and stiffness, as well as stability, of hydrogels. Nonetheless, the uncontrollable exchange of ions makes a brittle hydrogel [12]. To compensate for this issue, chemical crosslinking was used to create stable covalent bonds between the polymer chains. In this regard, different studies have focused on the modification of the carrageenan network with various natural and synthetic monomers. These modifications change the mechanical and biological properties of the hydrogel, leading to different applications. Chemical interactions with the secondary polymeric network, or various types of nanoparticles, are another promising approach to overcome the challenges facing carrageenan hydrogels [13,14,15,16,17].

There are various review articles focused on carrageenan-based biomaterials, and their pharmaceutical potential and biomedical applications [2,3,4,5,9,18,19,20,21]. In a complete review study, Yegappan et al. [9] discussed the different forms of carrageenan-based hydrogels and their biomedical applications. In another study, Necas et al. [5] reviewed the pharmacokinetics, toxicity, and biological potential of carrageenan. However, the presence of a review study focused on the different modification approaches of carrageenan, along with hybrid carrageenan-based platforms for biomedical applications, is still lacking. In this review, after the evaluation of the specific properties of carrageenan for biomedical applications, we will discuss different carrageenan modification approaches and hybrid carrageenan-based platforms that have been developed for various biomedical applications. Furthermore, the cytotoxicity and immunogenicity of carrageenan-based platforms are considered.

## 2. Carrageenan Properties for Biomedical Applications

Carrageenan shows potential bioactive properties for biomedical applications, consisting of biodegradation, biocompatibility, hemostatic ability, antioxidant, antitumor, and immunomodulatory properties, and protective activity against viral, bacterial, and fungal infections and influenza viruses such as dengue virus, herpes viruses, and hepatitis A virus [22,23,24,25]. Studies have reported the free-radical scavengers in vitro, and the antioxidant ability of carrageenan oligosaccharides and their derivatives in living organisms [25,26]. Many biological and photochemical reactions produce extremely toxic species such as superoxide anions [26]. Hydroxyl radical is a dangerous reactive oxidative species that formed through the disintegration of the superoxide anion and can quickly react with biomolecules, including amino acids, proteins, and DNA [27]. It was pointed out that the hydroxyl and amino groups in the polymer chains could take part in free-radical scavenging and antioxidant activity [28]. The hydroxyl groups in the κ-carrageenan polymer chain show antioxidant activity. The κ-carrageenan with a lower molecular weight generates short polymer chains after degradation with low contents of sulfate groups, leading to a reduction in the intramolecular hydrogen bonds. Consequently, the hydroxyl groups activate the hydrogen bonds, allowing them to easily react with the superoxide anions and hydroxyl radicals. During the degradation mechanism, the declined sulfate groups are replaced by hydroxyl groups, which means that the number of hydroxyl groups increases in degraded products. This procedure reveals the useful antioxidant activity of degraded κ-carrageenan in biomedical studies [26]. Various structural parameters, such as molecular weight, kind and situation of functional groups—hydroxyl, sulfate, and amine—affect the antioxidant ability of polysaccharides. At a lower molecular weight, due to the non-compressed structure of the polysaccharide and increased accessibility of hydroxyl and amine groups, the antioxidant capacity is stronger [29].

Many studies have also shown that three important types of carrageenan, i.e., κ-, ι-, and λ-carrageenan, have antiviral capacity [30,31,32]. Carlucci et al. [33] assessed the ability of carrageenan to promote the viability of Vero cells. They reported that carrageenan-based polymers did not show cytotoxic effects. They also revealed that λ-carrageenan showed more inhibitory behavior against drug-resistant viruses, while κ/ι carrageenan was slightly less efficient. Their result pointed out that the antiviral activity of sulfated polysaccharides has a relationship with molecular weight, the degree of sulfation, and the distribution of the sulfate groups of carrageenan. The antiviral mechanism in λ-carrageenan is explained by the fact that carrageenan occupies sites of the cell surface; thus, the virus can not bind to the cell and the cell is protected against the virus [34]. Carrageenan also demonstrates antibacterial effects against various bacteria. The antimicrobial capacity of polysaccharides on foodborne pathogenic bacteria was assayed by Yamashita et al. [35]. They represented that the maximum inhibitory effect is associated with carrageenan. It was also demonstrated that κ-carrageenan has good results in combination with commercial drugs, such as Flo CRS^®^ and Flo Sinus Care^®^ in the protection against Staphylococcus aureus infection [36].

The structure of carrageenan also has an important effect on the anticoagulant activity. The binding to various biologically active proteins can be improved by sulfate groups in the chemical structure and lead to anticoagulant ability. A high molecular weight and sulfate content in carrageenan can directly affect the anticoagulant activity [24]. For example, ι-carrageenan has demonstrated an anticoagulant capacity 3-fold stronger than κ-carrageenan. It was related to the greater number of sulfate groups in the ι-type [29]. In addition to the sulfate group content, the substitution position of the sulphate groups can affect this property. C-2 of 3,6-anhydro-α-d-Galp is probably the most attractive situation for replacement, whereas C-6 of β-d-Galp has no advantage. The anticoagulant effect of the sulphate groups at C-2 of the β-d-Galp units is more effective than the C4-sulphate samples [37]. Besides, carrageenan shows antitumor activities and few side effects [38,39]. The inhibitory activity of λ–carrageenan against the growth of the tumors is considerably dependent on the molecular weight. Zhou et al. [40] have shown that λ-carrageenans with different molecular weights have a great potential in the proliferation of nature killer (NK) and lymphocyte cells. Researchers believe that the ability of carrageenan oligosaccharides in the destabilization of the interaction between the glycosaminoglycans part of the proteoglycans and the extracellular matrix proteins results in eliminating the attachment of cancer cells to matrices, which is essential for the propagation of metastasis and antitumor activity [41]. The studies have confirmed the fact that the antitumor activity of carrageenan has a reverse relation with molecular weight. Moreover, high-sulfonated oligosaccharides show high antitumor properties compared to light-sulfonated or non-sulfonated samples [41,42]. Additionally, the ability of carrageenan-based hydrogels to impact the cytokine generation by cells greatly relies on the amount and structure of the polysaccharides [37]. Nevertheless, the relationship between the natural carrageenan chemical structure and biological property is still not thoroughly investigated. In a study by Yermak et al. [43], the effect of the structural peculiarities of carrageenan on its immunomodulatory and anticoagulant activities has been considered. Their study demonstrated that at high concentrations all types of carrageenan could enhance the level of pro-inflammatory interleukin 6 (IL-6) and tumor necrosis factor-alpha (TNF-α), while at low concentrations (1–10 ng/mL) their activity was insignificant. Additionally, it has been proved that all types of carrageenan could induce the secretion of anti-inflammatory interleukin 10 (IL-10) in a dose-dependent manner. The presence of an extra sulfate ester residue in λ-carrageenan escalated the amount of Ca in the macrophage cytoplasm, suggesting that it may contribute to the activation procedure of the production of active oxygen types. Furthermore, ι/κ-carrageenan had anticoagulant potential, which was remarkably high at low amounts. Carrageenans’ immunomodulation and anticoagulant behaviors are influenced by the monosaccharide structure of polysaccharides, as well as the placement, amount, and distribution of sulfate groups (SO_4_) along the galactan chain [43].

However, some drawbacks associated with carrageenan can affect their efficiency when used for bio-applications. For instance, the sulfate groups of carrageenan display adverse effects on blood coagulation and the immune system [3]. The anticoagulant activity of carrageenan correlates with the sulfate group contents, and the type of carrageenan should be selected according to its applications [44]. For example, carrageenan with fewer sulfate groups is more suitable for blood contact biomaterials, such as TE or DD carriers [9,45]. Besides, carrageenan can induce an inflammatory reaction for the investigation of anti-inflammatory drugs. Some studies reported that the long-term utilization of carrageenan in animals induced ulcerous colonists or damage to the mucous layer of the digestive system, and tumor growth [21,46]. Thus, more epidemiological and vital studies are needed to assess the safety of carrageenan [47].

## 3. Chemical Modification of Carrageenan

**Figure 1:**
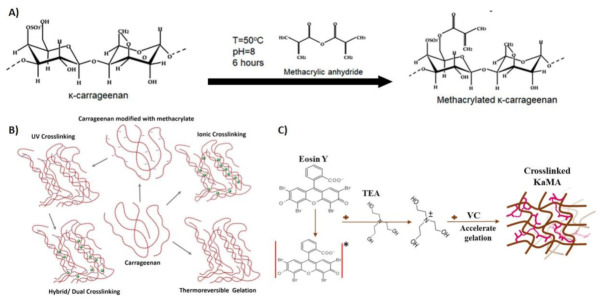
Methacrylate κ-carrageenan (KaMA) synthesis and hydrogel formation.: (**A**) Schematic representation of the synthesis methacrylate κ-carrageenan. Reproduced with permission from [10]. (**B**) Assessment of dual-crosslinked hydrogel. Reproduced with permission from [9]. (**C**) Visible light crosslinking approaches of κ-carrageenan methacrylate via eosin Y. Reproduced with permission from [14].

Polymers are usually treated with different chemical modification strategies, such as oxidation, methacrylation, esterification, thiolation, acetylation, and aldehyde-modification, in order to promote different properties [48]. Table 1 summarizes the various functionalization of the carrageenan backbone using various types of monomers for different biomedical applications. Between them, the methacrylation of carrageenan, and the synthesis of methacrylate κ-carrageenan (KaMA), has attracted wide attention in the recent years [10,11,13,14,49,50]. According to Figure 1A, by substituting the hydroxyl groups on κ-carrageenan with methacrylate groups, the methacrylation process occurred. The extent of the substitution of hydroxyl groups was considered equivalent to the degree of methacrylation [11,51,52]. Compared to other modification approaches, the methacrylation process provides the ability to be photocrosslinked in the presence of a chemical photoinitiator, including Irgacure 2959 [11,12,13,53,54], eosin Y [14,50], α-ketoglutaric acid [55], and LAP [56], under ultraviolet (UV) light [57] or even visible light [14]. The UV light photons lead to the segregation of the photoinitiator into two radicals, which cause radical formation on the methacrylate groups. The radicals quickly merge with each other nearby because they are unstable entities, which results in the formation of a crosslinked network [58,59]. The covalent bonds created throughout the crosslinking give the polymer network a lot of stability. The chemical modification, coupled with the already existing ionic character of κ-carrageenan, permitted the creation of double-crosslinked hydrogels through blending the chemical and physical crosslinking approaches. According to Figure 1B, the blending of the physical (K^+^) and chemical crosslinking process (UV exposure) resulted in strong ionic interactions between the sulfate groups and K^+^, while the formation of covalent bonds through methacrylate groups and photoinitiator makes the hydrogel more stable at physical conditions. These changes in the chemical structures resulted in the promoted integrity of KaMA with enhanced mechanical properties [9]. A higher degree of methacrylation and polymer concentration led to increasing covalent links through the higher density of photo crosslinkable units. Consequently, the compressive strength and moduli of the hydrogel increased [11].

To overcome the drawbacks associated with the UV crosslinking process, researchers have encouraged recruiting a safer wavelength of light, such as visible lights [72]. The use of visible light, with a lower energy than UV light, leads to the attenuation in DNA detriment and the elevation in cell viability opportunity [73]. Eosin Y, a photoinitiator activated with visible light, is a suitable substitution for a UV photoinitiator. According to Figure 1C, the interaction between the methacrylation groups and photoinitiators under visible-light exposure causes a visible light crosslinking process. Eosin Y, as a photoinitiator, motivates from the ground state to triple stat with visible light. In addition, visible light can abstract hydrogen atoms from triethanolamine as a co-initiator. The deprotonated triethanolamine (TEA) radicals then initiate the formation of a radical center on the methacryloyl groups. The visible-light gelation mechanism often entails co-monomers, such as *N*-vinylcaprolactam (VC), which raise the vinyl group concentration and increase the rate of the gelation procedure. The alteration in color from red to yellow for eosin Y confirms the photoinitiation and organization of activated eosin Y [58]. Recently, we synthesized a sprayable visible-light KaMA hydrogel [14]. Herein, eosin Y, TEA, and VC were added to the initial KaMA solutions, and the crosslinked structure with strong chain integration showed mechanical properties and a degradation rate suitable for the regeneration of target tissue.

**Figure 2:**
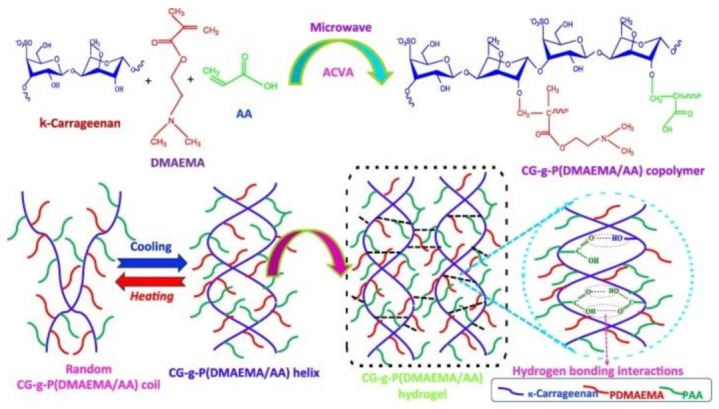
The chemical structure and architecture of carrageenan -g-P(DMAEMA/AA) copolymer. Reproduced with permission from [74].

In addition to the methacrylation process, other chemical modifications were also reported to control various mechanical, chemical and biological properties of carrageenan. For instance, Geyik et al. [74] synthesized a binary graft based on carrageenan and two monomers of dimethylaminoethyl methacrylate (DMAEMA) and acrylic acid (AA) using microwave irradiation. According to Figure 2, the synthesis of carrageenan, the copolymer, was made in a microwave oven. The particular concentration of carrageenan was dissolved in deionized water, and then various concentrations of 2-dimethylaminoethyl methacrylate and neutralized acrylic acid were incorporated into the solution, and the microwave irradiation power was implemented in the reaction. The grafting reaction was initiated by the addition of 4,4′-Azobis (4-cyanovaleric acid) (ACVA) to the solution, leading to the formation of the copolymer and changing the structure from helix to random coil. This copolymerization provided temperature/pH-sensitive swelling–deswelling transitions due to the presence of polyacrylic acid, which was grafted to carrageenan. They represented this hydrogel as an outstanding choice regarding controlled release purposes, employing the change in pH in the gastrointestinal system as a trigger regarding drug delivery.

**Figure 3:**
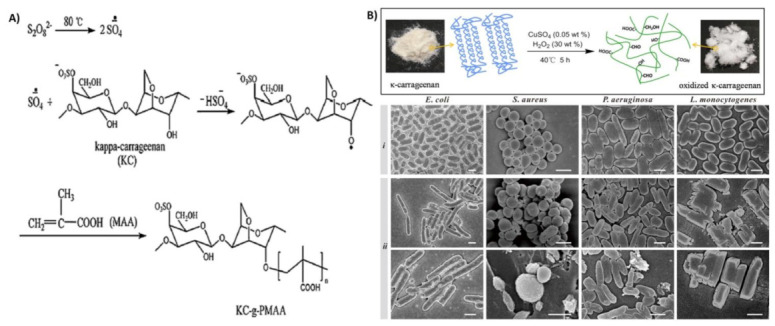
(**A**) The synthesis of copolymer κ-carrageenan-g-poly(methacrylic acid). Reproduced with permission from [77]. (**B**) The schematic representation of the modification of the structure of κ-carrageenan following specific oxidation and SEM images of bacteria (i) unmodified and (ii) modified with κ-Ox-3 at MIC value. Note: The scale bar is equivalent to 1 μm. Reproduced with permission from [69].

In another study, Kulkarni et al. [75] also synthesized a pH-responsive polyacrylamide-grafted κ-carrageenan. This copolymerization underwent ionization at a higher pH, leading to the maximum swelling and drug release in the intestine. In another study, Pourjavadi et al. [76] synthesized κ-carrageenan-grafted acrylic acid-co-2-acrylamido-2- methylpropanesulfonic acid (AA-co-AMPS) using a free-radical polymerization method. The crosslinking graft copolymerization was carried out using ammonium persulfate (APS) as a free-radical initiator and methylenebisacrylamide (MBA) as a hydrophilic crosslinker. The persulfate precursor was decomposed under heating to create sulfate anion radicals. Thereafter, the radicals extracted hydrogen atoms from the hydroxyl groups of the κ-carrageenan to form alkoxy radicals on the substrates, and they also made active centers on the substrate to radically initiate the polymerization of AA-co-AMPS, leading to a graft copolymer onto κ-carrageenan. This copolymerization deceased degradation and increased the swelling ratio (five-times bigger), and could be considered as an excellent candidate to design novel DD systems. In an interesting study, Chen et al. [77] developed a κ-carrageenan-g-poly(methacrylic acid)/poly(*N*,*N*-diethylacrylamide) copolymer hydrogel using APS as an initiator and *N*,*N*,*N’*,*N’*-tetramethylethylenediamide as an accelerator (Figure 3A). In this study, APS made active centers on the substrate and initiated copolymerization. The results showed improvement in the biocompatibility and thermo-sensitivity of the hydrogels, which could be expected to be useful for the DD system. Carboxymethylation is another modification of polysaccharides that can increase their properties. The existence of sulfate and carboxylate groups in carboxymethyl-kappa- carrageenan caused antibacterial activity due to an acidic pH ambience [63]. In a study, researchers used hydrogen peroxide (H_2_O_2_) and a copper sulfate (CuSO_4_) redox system for the modification of κ-carrageenan (oxidized κ-carrageenan(κ-Ox-3)), and they investigated its effect on the antibacterial ability (Figure 3B). They confirmed that oxidized κ-carrageenan destroyed the cytoplasmic membrane of both Gram-positive and Gram-negative bacteria (Figure 3B(i,ii)) [69].

## 4. Hybrid Carrageenan-Based Platforms and Its Application 

Carrageenan-based hydrogels have been applied for various biomedical applications, including TE, DD, and wound healing. Various chemical modification strategies could significantly change the mechanical and biological properties of carrageenan. Consequently, depending on the target application, various chemical modifications have been explored on the carrageenan chains. In addition, different types of nanoparticles have been incorporated into carrageenan-based hydrogels. Accordingly, here we explored various hybrid carrageenan-based hydrogels for different applications.

### 4.1. Hybrid Carrageenan Based Platforms for Tissue Engineering

Carrageenan has been explored over the recent years as a promising candidate in TE and regenerative medicine. According to the mechanical properties of hybrid hydrogels, they can be applied for hard or soft TE. These hydrogels are usually incorporated with other types of polymers or nanoparticles to improve the properties of hydrogels for target tissue regeneration. Table 2 presents various applications of these hybrid hydrogels for TE.

#### 4.1.1. Hard Tissue

Hard tissues include bone and tooth structures that contain both organic and inorganic components, most are collagen type I and calcium-phosphate minerals [90]. Carrageenan-based hydrogels reveal the potential for bone tissue regeneration due to their ability to induce bone-like apatite formation [91]. However, the mechanical properties of carrageenan should be promoted to stimulate the mechanical properties of hard tissue. Between them, ι- carrageenan-based hydrogels show a higher viscosity compared to κ-carrageenan, and they are suitable for hard tissue regeneration. For instance, Ashe et al. [86] prepared gelatin/ι- carrageenan/silk blended hydrogels and investigated their physicochemical properties for bone regeneration. The hydrogels showed appropriate bone-specific cell attachment, proliferation, and survival. In another research, Yegappan et al. [87] designed injectable ι- carrageenan nanocomposite hydrogels encapsulated within whitlockite nanoparticles and an angiogenic drug. Osteogenic differentiation in rat adipose-derived mesenchymal stem cells after 14 days increased the degrees of alkaline phosphatase activity in vitro. In addition, human umbilical vein endothelial cells (HUVECs) created capillary tube-like structures after being exposed to the modified hydrogel. The nanocomposite hydrogel had angiogenic and osteogenic characteristics that might have essentially harnessed in bone TE.

Conductive hydrogels have also been developed by the modification of the carrageenan hydrogel using gold nanoparticles (NPs), which present biological cues for cell–matrix interactions for bone reconstruction. In another study, Pourjavadi et al. [89] synthesized a chitosan/κ-carrageenan hydrogel modified with gold NPs. Interestingly, the improved proliferation and attachment of MG-63 cells was a result of the enhanced conductivity of the scaffold by the addition of gold nanoparticles. Gold NPs in the backbone of the hydrogel work as electrical couplers between the cells and boost the electrical signal transfer between the cells and the neat scaffold. According to the few mechanical properties of κ-carrageenan hydrogels, they have not been widely applied for bone TE.

#### 4.1.2. Soft Tissue

**Figure 4:**
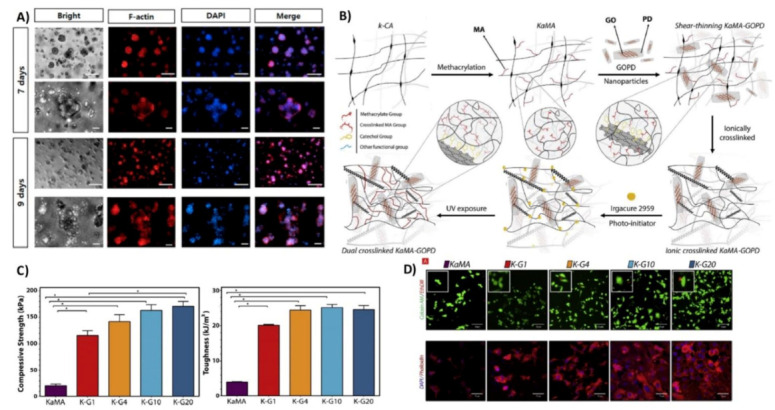
Hybrid carrageenan-based system for soft TE.: (**A**) 3D incorporating NIH-3T3 cells in carrageenan hydrogel assay with DAPI/F-actin staining image at days seven and nine. Reproduced with permission from [10]. (**B**) Schematic outlining the design strategy to synthesize nanohybrid KaMA–GOPD hydrogels. (**C**) Mechanical properties of KaMA–GOPD hydrogel through compressive strength (at 60% strain) and toughness. (**D**) Fluorescence images of live/dead assay of fibroblasts seeded on the KaMA–GOPD hydrogels at day five of incubation. (Calcein AM (green) and EthDII (red) presented live and dead, respectively). Reproduced with permission from [13].

Carrageenan-based hydrogels have also recently been applied for soft TE. For instance, İlhan et al. [49] used a microwave-methacrylated κ-carrageenan hydrogel as a bioink for cartilage regeneration. They demonstrated that the utilization of microwave energy made the methacrylation process more efficient and increased the number of crosslinking sites available, leading to the boosted chemical photocrosslinking reactions. This significantly enhanced the strength of the hydrogels through increasing chain entanglements during photopolymerization, and consequently the stiffness of the hydrogel improved dramatically. The modified hydrogels, with increased material stiffness and degradation resistance, escalated ATDC5 cell attachment, spread, and differentiation. In an interesting study, Thakur et al. [12] reinforced the chemical crosslinked κ-carrageenan using 2D nanosilicates for the encapsulation of human mesenchymal stem cells (hMSCs) and cartilage TE. The addition of nanosilicates to κ-carrageenan resulted in the development of shear-thinning features, implying the potential of nanosilicates to interact strongly with κ-carrageenan polymer chains and render injectability to prepolymer solutions for cellular delivery. The nanosilicates additionally strengthened the κCA hydrogel network to boost the physiological stability and elastomeric properties. The addition of nanosilicates to the KaMA hydrogel resulted in a flexible network, which improved the mechanical stiffness as well as the elastic characteristics of the gels at higher strains. Due to ability of nanosilicates to interact strongly with KaMA polymer chains, the nanocomposite hydrogel shows a higher compressive modulus and a more effective crosslinking mechanism compared to either hydrogel without the nanoparticle. Cross et al. [54] displayed a gradient scaffold with two natural polymers, gelatin methacryloyl and methacrylated κ-carrageenan, encapsulated with 2D nanosilicates to imitate the native tissue interface. The effective cell incorporation of hMSCs and the regulation of cell morphology exhibited the ability to direct cell fate throughout the network and likely direct cell differentiation without the utilization of growth factors. This hydrogel might be employed to reconstruct the bone–cartilage interface, where a natural gradient in the structural, mechanical, and cell interaction is present. Lim et al. [10] applied a dual-crosslinkable bionic using methacrylated κ-carrageenan for soft TE. Bioprinting using the cell-laden (NIH-3T3 cells) hydrogel showed cell compatibility with an enhanced shape retention capability. The hydrogel showed great potential as a bioink candidate for the bioprinting of soft tissues. Also, Yu et al. [79] reported a facile approach to prepare *κ*-carrageenan/chitosan hydrogels as structural biomaterials with a great self-recovery capacity, cytocompatibility, and cell anti-adhesion characteristic. The encapsulation of multiple noncovalent interactions improved the toughness of the biopolymer-based hydrogels. The hydrogel was proposed for an artificial dura mater and diaphragm materials in surgery. In another research, Rode et al. [81] used the κ-carrageenan hydrogel as a scaffold for the in vitro culture of human skin-derived multipotent stromal cells. Skin-derived multipotent stromal cells cultured inside the carrageenan hydrogels presented as spherical, and retained their viability and spread for a week of incubation (Figure 4A). They proved the potential use of the carrageenan hydrogel as a cell carrier in skin regeneration. Tavakoli et al. [14] developed a sprayable visible-light crosslinked KaMA hydrogel to cover skin injuries or inject as a bioprinting material to in situ heal soft tissue damages. The bonding strength of the hydrogel was higher compared to commercially accessible tissue adhesives, and might enhance the adhesion and spread of cells in vitro. Also, they demonstrated that a higher methacrylation degree led to a denser crosslinked structure with improved mechanical strength and moduli. Consequently, the covalently crosslinked hydrogel can absorb higher energy, which improves the toughness. Recently, Mokhtari et al. [13] fabricated a dual-crosslinking hydrogel based on KaMA and dopamine-modified modified graphene oxide (GOPD) for soft TE and 3D bioprinting. Figure 4B shows how the GOPD interacted in the polymeric network in order to make a nanocomposite dual-crosslinking hydrogel. Besides the ionically and covalently crosslinking processes, the chemical interaction between the catechol groups of dopamine with other moieties on KaMA was provided, leading to an improvement in the KaMA hydrogel properties. The hybrid hydrogel revealed great shear-thinning behavior and injectability through the interaction of nanoparticles with other moieties of the polymer. The mechanical results also showed that with the addition of GOPD to the polymeric network, the mechanical strength improved significantly (Figure 4C). This demonstrated the role of nanoparticle interaction on mechanical properties and how it makes the crosslinking process more efficient. This also increased the ability of the hydrogel to absorb energy and, as a result, the toughness of the nanocomposite enhanced. Also, they found out that with the addition of the nanoparticle there was an increase in the adhesion sites to attract proteins inside the culture media through electrostatic interactions, leading to improved cell attachment and spreading (Figure 4D).

Another promising strategy is the blending of several types of polymer networks to provide specific applications. For instance, Tytgat et al. [56,60] established a hybrid hydrogel based on methacrylamide gelatin (GelMA) and KaMA hydrogels for the regeneration of women’s soft tissue with breast cancer. The hydrogel blend was able to absorb large amounts of water and exhibited mechanical properties comparable to native breast tissue. Adipose tissue-derived stem cells seeded on hydrogel showed good cell viability and proliferation. In other research, Kim et al. [82] developed alginate/κ-carrageenan hydrogels using extrusion-based 3D bioprinting. Adipose-derived mesenchymal stem cells were used to evaluate the cytocompatibility of bioink. The results demonstrated the suitable ratio of alginate/κ-carrageenan, presenting the possibilities to turn them into a potential bioink for manufacturing an appealing 3D-printed scaffold with the outstanding mechanical performance, whilst sustaining the structure and biological properties in the area of the soft TE. Akrami et al. [83] also used a combination of chemical modification, nanoparticle incorporation, and blending with other polymer networks to overcome the gelatin and halloysite nanotubes to simultaneously improve the mechanical properties and cell functions. Araujo et al. [80] made a polyelectrolyte blend of chitosan/carrageenan. This polyelectrolyte is formed by reversible ionic condensation between a positively charged polyelectrolyte (chitosan) and a negatively charged one (κ-carrageenan). The results proved that the chitosan/carrageenan blend exhibited large and interconnected pores that were stable at acidic and neutral pH, and were observed to dissolve at pH 9 and 11. This gave the scaffold the ability to seem appealing for TE-associated applications that need the use of pH-sensitive components stable at physiological conditions. Liang et al. [78] proved that the chitosan/carrageenan composite hydrogels displayed a homogeneous multiple-crosslinked network structure, depending on the physical crosslinking created via hydrogen bonds and ionic bonds between the two polyelectrolytes. They proved to have excellent mechanical properties and biocompatibility. This is important in biological applications in terms of cartilage repair. In an interesting study, Li et al. [85] applied two oppositely charged hydrogels, including anionic κ-carrageenan and cationic GelMA, to develop 3D printed structures with improved adhesion between the layers for soft TE. This polyelectrolyte complex was also applied for the encapsulation of C2C12 cells, and the results confirmed that a viability more then 98% was achieved.

### 4.2. Hybrid Carrageenan Based Platforms for Wound Healing

**Figure 5:**
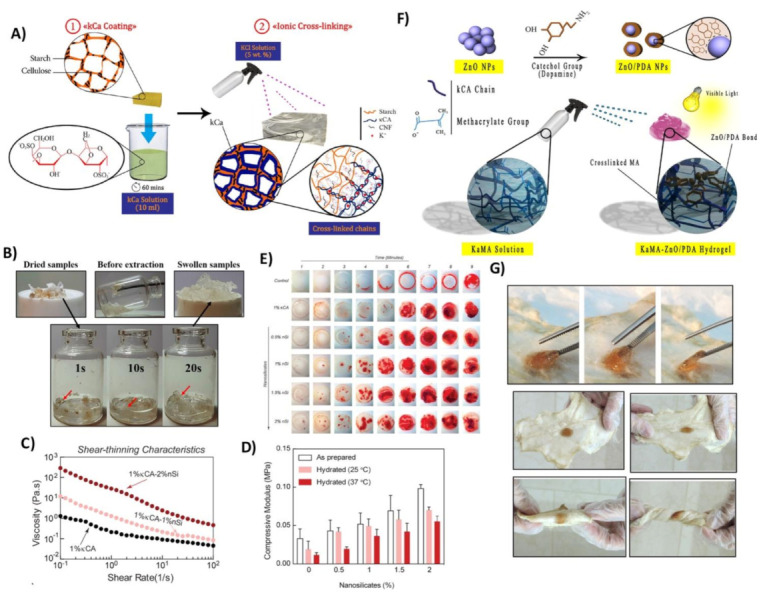
Hybrid carrageenan-based platforms for wound healing applications. application: (**A**) Schematic of the two-step synthesis process of κ-carrageenan-coated starch/CNF (**B)** photograph images of the coated sample during the swelling evaluation, demonstrating the high swelling ability of nanocomposite hydrogel. Reproduced with permission from [15]. (**C**) The shear-thinning characteristics of pre-polymer hydrogels representing that the incorporation of nanosilicates escalated viscosity at various shear rates. (**D**) Nanosilicates incorporation reinforced the mechanical stiffness of κ-carrageenan hydrogels. (**E**) Clotting kinetics of whole blood with respect to time and nanosilicate concentration in κ-carrageenan hydrogels. Reproduced with permission from [103]. (**F**) The schematic illustration NPs modification and their interaction with KaMA hydrogel, before and after visible light (chemical) crosslinking. (**G**) Tissue-adhesion strength evaluation of Z/P hydrogels adhered to cow skin as a function of various deformations and images of peeling hydrogel from skin. Reproduced with permission from [50].

The skin is the largest organ of the human body, and it has a critical role in protecting the body against the external environment [92]. Although the skin possesses a high self-regeneration potential, severe skin injuries cannot heal spontaneously and need to be covered by wound dressings or skin substitutes [93]. In recent years, a tremendous effort has been made in the field of skin TE to produce wound coverage and skin grafts. Among the different types of materials, hydrogels are among the most suitable candidates with the highest possibilities to imitate the native skin medium because of their porous and hydrated molecular structure [16]. Numerous studies proved that natural-based hydrogels could provide a suitable platform or environment for efficient wound healing processes compared to traditional wound dressings, including bandages, cotton wool, and gauzes [9,16,94]. The majority of natural hydrogels are biocompatible, and do not provoke any adverse reaction and rejection upon implantation. Additionally, they can provide a suitable matrix with an optimum moisture amount, evaporation, and degradation rate to support cell functions including proliferation, migration, and re-epithelization [93]. Among the different natural-based hydrogel dressings accessible these days, hydrogels encapsulating natural polysaccharides are well known due to their ability to appear like glycosaminoglycans (GAGs), and their organization, convenience, user-friendliness, and affordability [95,96]. Carrageenan hydrogels are polysaccharide-based materials utilized for wound healing applications as they mimic the natural microenvironments, enabling improved cell–cell and tissue interactions [13,14,16]. However, carrageenan exhibits adverse effects on blood coagulation and the immune system due to the sulfate groups. However, it can be safely used in biomedical applications after carefully tailoring the sulfate groups. Furthermore, low mechanical strength, weak stability, and a high degradation rate in physiological conditions are still associated with bare carrageenan-based hydrogels, which limit their applications as a wound dressing. Therefore, their mechanical performance, including their stiffness, viscoelastic behavior, and initial state recovery (self-healing), and their biological properties for effective wound healing, can be tuned by chemical modification, crosslinking, copolymerization, and nanoparticle or biomolecules incorporation [93]. For example, it has been shown that the incorporation of cyclic β- (1-3) (1-6) glucan into ι-carrageenan hydrogels could increase fibroblast migration and accelerate the wound healing process in vitro and in vivo, with notable antibacterial activity against *S. aureus* [97]. Although λ- and ι-carrageenans have been employed to synthesize wound dressings [61,97,98,99,100,101], the majority of carrageenan-based wound dressings have been synthesized from κ-carrageenan as it contains the lowest sulfate content and it can form the strongest gel involving a coil to helix conformational transition followed by helix aggregation [5,102]. Table 3 presents recent studies on the κ-carrageenan-based hydrogels applied for wound healing applications. In a recent study, we applied a modified κ-carrageenan hydrogel to the surface of a starch/cellulose nanofiber (starch/CNF) using a new approach. We aimed to simultaneously improve the swelling ratio, mechanical properties, and hemorrhage ability (Figure 5A) [15]. Our results illustrated that this hydrogel had a considerable swelling ability with optimum mechanical strength and stability, which can be placed at the wound site and stop bleeding (Figure 5B).

The incorporation of nanoparticles into the κ-carrageenan matrix to synthesize nanocomposite hydrogels has offered some unique characteristics to the wound dressings, including robust mechanical properties, controllable degradation rate, optimum swelling ratio, viscoelastic manner, shear-thinning behavior for spraying/injecting, antibacterial activity, cell function modulation, and wound healing acceleration. Lokhande et al. [103] have provided an injectable hydrogel for wound healing applications using κ-carrageenan and two-dimensional (2D) nanosilicates with the ionic crosslinking mechanism. Their findings indicated that this nanocomposite hydrogel is shear-thinning, allowing the wound dressing injection into the wound site with a non-invasive approach (Figure 5C). Additionally, the mechanical stiffness of this gel could be tuned by controlling the ratio between the κ-carrageenan and nanosilicates between 20 and 200 kPa, which is appropriate for skin applications (Figure 5D). Moreover, this engineered nanocomposite hydrogel could increase protein adsorption and consequently enhance cell adhesion and spreading, platelets binding, and reducing the blood clotting time (Figure 5E). Despite their notable results, this nanoengineered gel was not evaluated in terms of antibacterial activity, which is a critical issue in chronic wounds. Moreover, the in vivo performance of this hydrogel was not investigated. In our recent complementary study, we incorporated polydopamine-modified ZnO (Z/P) nanoparticles and L-glutamic acid into the KaMA hydrogel to accelerate diabetic wound healing (Figure 5F) [15,50]. Importantly, the hydrogel was shear-thinning even after the incorporation of the nanoparticles, allowing sprayability to this gel. Our results also illustrated that the addition of Z/P nanoparticles into the hydrogel matrix boosted the gel tensile and compressive mechanical properties. In addition, the nanocomposite hydrogel had a controllable degradation rate in the physiological environment and it could tightly adhere to the skin, and thereafter peeled off easily without any further injury (Figure 5G). Moreover, the results of the antibacterial evaluation demonstrated that this gel had a noticeable ability to prohibit bacterial activity and consequently prevent wound infection. Finally, the in vitro cell culture and in vivo animal study confirmed that the presence of the Z/P nanoparticles and L-glutamic acid could effectively increase cell proliferation, migration, and collagen synthesis, which eventuates in accelerated wound contraction and healing.

### 4.3. Hybrid Carrageenan Based Platforms for Drug Delivery

Carrageenan is presented as a gold candidate in pharmaceutical and biotechnological applications because of its biocompatibility, high viscosity, and gelling capacity, and the ability for its controlled release of drugs [9,34,116]. However, the fast degeneration and quick swelling of carrageenan cause the fast release of drugs with less effectivity [117]. In this regard, the interaction of different types of carrageenan with each other or other polymers is helpful to achieve ideal drug release platforms [118]. In a study, different types of carrageenans (κ-, ι-, λ-) were combined with chitosan at charge ratios of three and five to present an electrostatic interaction between the adversely charged SO_4_ groups in carrageenan and the positively charged amine groups (NH_3_) in chitosan. This platform was used for the controlled release of glucose oxidase (GOD). The encapsulation functionality of GOD in the chitosan/carrageenan complexes was greater compared to the neat polymer. Chitosan/κ-carrageenan complexes point out the greatest incorporation functionality accompanied by chitosan/ι–carrageenan and chitosan/λ–carrageenan at both charge ratios of three and five. The λ-carrageenan was the most anionic due to its three sulfate groups that affected the encapsulation efficiency of the complex. The GOD release rate was the lowest in the chitosan/κ-carrageenan complex because the κ-carrageenan possessed a SO_4_ group connected to its changing sugar units, fewer electrostatic repulsions were anticipated with a lesser number of ionic binding sites. The λ-carrageenan experienced the maximum release rate of GOD. This kind comprises three SO_4_ groups of the changing sugar units, generating more electrostatic repulsion and ionic binding sites [119]. Guzman-Villanueva et al. [120] developed the curcumin-loaded chitosan nanoparticles and incorporated them into the alginate–carrageenan hydrogel microparticles to escalate the solubility and stability of curcumin in gastrointestinal circumstances. These nano-microparticle carriers caused the release of over 95% of the loaded curcumin during 7 h of incubation in a pH 7.4 buffer solution, using an alginate/κ-carrageenan ratio of 50:50. Another study was conducted by Zhao et al. [121], who investigated the physicochemical properties and drug release ability of agar/κ-carrageenan hydrogels. The increased κ-carrageenan concentration in the agar/κ-carrageenan hydrogel decreased the gel strength and gelling temperature while it increased the viscosity. Metformin hydrochloride (MET) was used as a drug model, and the encapsulation efficiency and sustained release ability of agar hydrogels were evaluated. The negatively charged agar and carrageenan molecules could be attracted to the positively charged MET, which proves the high encapsulation efficiency of MET. The sustained release of the drug was shown until 9 h via the encapsulation of κ-carrageenan. The release pattern was primarily dominated by the electrostatic interaction between the polysaccharides and the drug. Graf et al. [122] synthesized a nasal spray with iota-carrageenan and xylometazoline HCl for the improvement of nasal obstruction caused by sinusitis. The removal property of the nasal obstruction related to the drug and antiviral capacity of iota-carrageenan were among the reported results of the in vitro experiment. Morokutti-Kurz and coworkers [123] combined iota- and kappa-carrageenan and incorporated zanamivir as an anti-influenza drug, and they demonstrated that a combination of different types of carrageenan could result in significant prevention against several influenza A viruses (pandemic H1N1/09, H3N2, H5N1, H7N7) in vitro and in vivo. Chiu et al. [124] prevented the human enterovirus 71 (EV 71) infection using κ–carrageenan virus complexes, in which kappa-carrageenan attached to the virus preventing it from attaching to the cell.

Carrageenan is a superior polymer to deliver antitumor drugs for oral chemotherapy due to the carrageenan oligosaccharides that elevate the immune system and, therefore, the antitumor capacity [25]. Nanostructured lipid–carrageenan hybrid carriers (NLCCs) were employed regarding the controlled delivery of chemotherapeutic agents, including mitoxantrone hydrochloride (MTO) with a great loading efficiency, sustained-release pattern, and possibilities for enhancing oral bioavailability and antitumor effectiveness. Regarding the MTO solution, an estimated 98.9% of free MTO was released after 4 h, although 92.3% of the MTO was released from the NLCs after 12 h. It was identified that just 79.6% of the MTO was released from the NLCCs after 48 h, resulting in the sustained-release characteristics of MTO because of the exerting of carrageenan. The electrostatic interaction played a major role in sustained-release behavior. The oral bioavailability of MTO loaded in NLCCs was considerably boosted due to the endocytic process of the NLCCs that escaped the BCRP-mediated efflux. The cytotoxicity results confirmed that the NLCCs could remarkably improve the antitumor efficacy against MCF-7/MX cells [125]. In another study, a biocompatible and biodegradable DD system based on a κ-carrageenan-grafted GO nanocarrier was suggested for anticancer DD. This nanocarrier was further conjugated with biotin to achieve targeted drug delivery. Doxorubicin (DOX) was used as an anticancer drug for any kind of cancer, and was entrapped over on the GO surface. The in vitro drug release pattern of DOX was carried out at various pH, including pH 2.8, pH 5.5, and pH 6.8 in the physiological medium at 27 °C for 24 h. The sustained release of DOX was obtained at pH 5.5 (50%) and pH 6.8 (64%) during the 24 h. Under acetic conditions (pH 2.8), a significant amount of the drug (74%) was released. The GO/κ-car–biotin nanocarriers indicated high levels of cell death of the cancer cells, which proved the targeted DD of these nanocarriers [126]. Despite the intrinsic antibacterial properties of κ-carrageenan, controlled release of antibacterial agents has still been performed for the improvement of bacterial prohibition.

## 5. Conclusions and Future Perspective

Here, we reviewed various applications of carrageenan in drug delivery, wound healing, and TE. To provide the desired properties for these applications, carrageenan-based hydrogels should be modified using various functional groups. Noticeably, the development of photocrosslinking methacrylate κ-carrageenan (KaMA) hydrogels accelerated to ability to overcome the weak mechanical properties and improve the physiological instability of carrageenan hydrogels. In addition, the modulation of methacrylation degree, crosslinking density and polymer concentration are the main tools for controlling the mechanical properties and, consequently, the cell behavior in response to the KaMA hydrogel. These parameters and their modulation give researchers different choices to make tailored carrageenan hydrogels for mimicking the behavior of different tissues. Moreover, various types of nanoparticles and polymers have been applied to develop hybrid carrageenan platforms. These platforms can simultaneously improve mechanical and physical properties, and specifically the biological characteristics of carrageenan hydrogels, depending on the secondary component composition. Chemically modified and hybrid carrageenan-based hydrogels can provide three-dimensional structuring mimicking the native extracellular matrix, making them appropriate for cell and bioactive molecule delivery.

However, the path is outlined and currently the development of new studies focused on the uses of carrageenan is booming. The future of carrageenan-based hydrogels is placed at the extremes of balancing their functional therapeutic requirements and functions, their accessibility in relatively greater quantities, and the obvious comprehending of their individual physiochemistry. Fascinatingly, the biological activities of carrageenan-based hydrogels have commonly revealed eye-catching biological activities, but understanding the relationship between the individual structures and biological properties is still the main challenge in the studies. To overcome these challenges, we propose that the modulation of chemical properties and structural properties should be performed to assess the interaction between biological components and hydrogels. Besides, the inadequate knowledge on the mechanistic pathways of the DD from carrageenan-based hydrogels also points in the direction of further analyses in the future. In addition, most of the scientific reports focus mainly on in vitro biological studies, and the correlation studies for in vivo and in vitro–in vivo are fairly restricted. However, the different therapeutic applications are still in the experimental phase as there are certain limitations in the use of these hydrogels for biomedical applications. In addition, deeper scientific studies are necessary for understanding the role of κ-carrageenan-based hydrogels on the controlled release of various therapeutic agents, applied in wound dressing and tissue engineering.

## Figures and Tables

**Table 1:** Recent advances in different chemical modifications of carrageenan.

Carrageenan Type	Chemical Modification	Target Application	Ref.
κ-carrageenan	Methacrylation	Cell–material platforms for TE	[11]
κ-carrageenan	Methacrylation	Bioink for cartilage TE	[49]
κ-carrageenan	Methacrylation	Sprayable hydrogel to cover skin injuries and heal soft tissue damages, bioadhesive hydrogels for chronic diabetic wound healing	[14,50]
κ-carrageenan	Methacrylation	Injectable shear-thinning and mechanically robust hydrogel for soft TE	[13]
κ-carrageenan	Methacrylation	Shear-thinning hydrogels can be used for cell delivery for cartilage tissue regeneration	[12]
κ-carrageenan	Methacrylation	Hydrogel for adipose TE, 3Dscaffolds for the differentiation of adipose tissue-derived stem cells into the adipogenic lineage	[56,60]
κ-carrageenan	Methacrylation	Gradient scaffolds for mimicking tissue interfaces and cartilage tissue regeneration	[54]
κ-carrageenan	Methacrylation	Injectable delivery vehicle for wound healing and tissue repair	[61]
κ-carrageenan	Methacrylation	Bioink for soft TE	[10]
κ-carrageenan	De-esterification	Examine the effect of de-esterification on κ-carrageenan gels	[62]
κ-carrageenan	Carboxymethylation	Biomaterials for cell-contacting applications in wound dressings	[63]
ι- carrageenan	Carboxymethylation	Carrier for the oral delivery of insulin	[64]
ι- carrageenan	Carboxymethylation	Nanocarrier system for the treatment of intracellular *C.glabrata* infections	[65]
κ-carrageenan /ι- carrageenan	Thiolation	Characterize a thiolated carrageenan as a novel pharmaceutical excipient	[66]
κ-carrageenan	Acetylation	Biomaterials for potential applications as anti-influenza virus	[67]
κ-carrageenan	Phosphorylation	Nanoparticles are a pretty system for simultaneous release of rifampicin and isoniazid in the treatment of tuberculosis	[68]
κ-carrageenan	Oxidation	Antibacterial agent against Gram-positive bacteria (*S. aureus* and *L. monocytogenes*) and Gram-negative bacteria (*E. coli* and *P. aeruginosa*)	[69]
κ-carrageenan	Oxidation	Evaluate the effect of oxidation on the anticoagulant activity	[70]
κ-carrageenan /ι- carrageenan	Cationization	Cationic polysaccharides for various aplications	[71]

Tissue engineering (TE).

**Table 2:** A summary of recent studies on carrageenan-based hydrogels for TE applications.

Hydrogel	Nanoparticle	Crosslinking	Tissue	Ref.
KaMA	-	Chemical crosslinking	Cartilage	[49]
KaMA	2D nanosilicates	Ionic and Chemical crosslinking	Cartilage	[12]
Gelatin methacryloyl/KaMA	2D nanosilicates	Chemical crosslinking	Cartilage	[54]
κ-carrageenan/chitosan	-	Chemical crosslinking	Cartilage	[78]
κ-carrageenan/chitosan	-	Ionic crosslinking	Connective tissue	[79]
κ-carrageenan/chitosan	-	-	Soft tissue	[80]
KaMA	-	Ionic and Chemical crosslinking	Soft tissue	[10]
κ-carrageenan	-	Ionic crosslinking	Soft tissue (skin)	[81]
KaMA	-	Ionic and Chemical crosslinking	Soft tissue (skin)	[14]
KaMA	Dopamine functionalized graphene oxide	Ionic and Chemical crosslinking	Soft tissue	[13]
Methacrylamide- gelatin/KaMA	-	Chemical crosslinking	Soft tissue (adipose)	[56,60]
κ-carrageenan/alginate	-	Ionic crosslinking	Soft tissue	[82]
Aldehyde-modified κ-carrageenan/Gelatin	Halloysite nanotubes	-	Soft tissue	[83]
κ-carrageenan/sorbitol/glycerin	-	Ionic crosslinking	Soft tissue	[84]
κ-carrageenan/GelMA		Chemical crosslinking	Soft tissue	[85]
ι- carrageenan/Gelatin/Silk	-	-	Bone	[86]
ι- carrageenan	Whitlockite nanoparticles	Ionic crosslinking	Bone	[87]
Chitosan/κ-carrageenan	Hydroxyapatite nanoparticles	Ionic crosslinking	Bone	[88]
Chitosan/κ-carrageenan	Gold nanoparticle	-	Bone	[89]

**Table 3:** The summary of recent studies that applied κ-carrageenan for wound healing application.

Major Material	Major Findings	Ref.
Polyethylene oxide + κ-carrageenan + streptomycin + diclofenac	Excellent transparency, protection of the wound, controlled release of both streptomycin and diclofenac, antibacterial activity	[104]
κ–carrageenan + poly vinyl alcohol + Lactobacillus bulgaricus extract	Anti-inflammatory ability, antibacterial activity, accelerate the healing process of the chronic wound	[105]
κ–carrageenan + nanosilicates + vascular endothelial growth factor	Injectable hydrogel, increase platelets binding and reduce blood clotting time, facilitate wound healing in vitro	[103]
κ–carrageenan + Skin-derived stromal cells	Reduce inflammatory process, fast initial recovery of wounded area, improved extracellular matrix deposition	[81]
Astaxanthin + alpha-tocopherol + κ-carrageenan nanoemulsion	Biocompatible in vitro and in vivo, reduce fasting blood glucose levels and improve glucose tolerance, accelerate wound closure	[106]
Octenidine dihydrochloride + Chitosan-treated serum + κ-carrageenan	Injectable hydrogel, biocompatible gel in vitro, induce migration of polymorphonuclear neutrophils and fibroblasts, antibacterial activity	[107]
κ-carrageenan + chitosan capped sulfur NPs + grapefruit seed	Strong antibacterial activity, ultraviolet barrier property, efficient wound healing in vivo, complete appearance of the healed epidermis	[108]
κ–carrageenan + locust bean gum + cranberry extract	Dose-dependent cytotoxicity against NIH 3T3 fibroblast cells Provide a visual system for monitoring bacterial wound infections	[109]
ι-carrageenans + κ-carrageenan + locust bean gum + gelatin	Injectable hydrogel, biocompatible with good cell adhesion in vitro, able to release encapsulated growth factor to promote cell migration	[61]
κ-carrageenan+ pigmented protein C-phycocyanin	Injectable hydrogel, enhance proliferation of dermal fibroblasts in vitro without inducing inflammation, reduce the blood clotting time	[110]
Aldehyde-modified κ–carrageenan + gelatin + halloysite nanotubes	Biodegradable and biocompatible	[83]
κ–carrageenan +Agar + montmorillonite	Control drug release, antibacterial activity	[111]
κ–carrageenan + chitosan	Promote thrombin formation and hemostasis, promote tissue growth	[112]
κ–carrageenan + Ag-ZnO@ carboxymethyl cellulose + graphene oxide	Improve epithelialization, advance fibroblast development, quicken wound recuperating	[113]
κ–carrageenan + Z/P + L-glutamic acid	Antibacterial activity, reduce clotting formation time, accelerate wound contraction	[50]
κ–carrageenan+ starch/CNF	Superabsorbent ability, reduce clotting formation time	[15]
κ/β-carrageenan	Promote the secretion of anti-inflammatory factors and accelerate polarization, accelerate the repair process of the full-thickness excisional wound, improve collagen deposition	[114]
κ–carrageenan + Na-alginate + silver NPs	Control drug release, antibacterial activity	[115]

## Data Availability

All the relevant data used in the study have been provided in the form of figures and tables in the published article, and all data provided in the present manuscript are available to whom it may concern.
